# Potentiating Salvage Radiotherapy in Radiorecurrent Prostate Cancer Through Anti-CTLA4 Therapy: Implications from a Syngeneic Model

**DOI:** 10.3390/cancers16162839

**Published:** 2024-08-14

**Authors:** Hanzhi Wang, Linsey Gong, Xiaoyong Huang, Stephanie D. White, Hans T. Chung, Danny Vesprini, Tera N. Petchiny, Emmanouil Fokas, Hansen He, Robert S. Kerbel, Stanley K. Liu

**Affiliations:** 1 Department of Medical Biophysics, University of Toronto, Toronto, ON M5S 1L7, Canada; linsey.gong@mail.utoronto.ca (L.G.); steph.white@mail.utoronto.ca (S.D.W.); hansenhe@uhnresearch.ca (H.H.); robert.kerbel@sri.utoronto.ca (R.S.K.); stanley.liu@sunnybrook.ca (S.K.L.); 2 Sunnybrook Research Institute, Toronto, ON M4N 3M5, Canada; xiaoyong.huang@sri.utoronto.ca (X.H.); danny.vesprini@sunnybrook.ca (D.V.); tera.petchiny@mail.utoronto.ca (T.N.P.); 3 Sunnybrook Health Sciences Centre, Odette Cancer Centre, Toronto, ON M4N 3M5, Canada; hans.chung@sunnybrook.ca; 4 Department of Radiation Oncology, University of Toronto, Toronto, ON M5S 1P5, Canada; 5 Department of Laboratory Medicine and Pathobiology, University of Toronto, Toronto, ON M5S 1A8, Canada; 6 Department of Radiation Oncology, CyberKnife and Radiation Therapy, Centre for Integrated Oncology Aachen Bonn Cologne Duesseldorf (CIO ABCD), Faculty of Medicine, University Hospital of Cologne, University of Cologne, 50937 Cologne, Germany; emmanouil.fokas@uk-koeln.de; 7 Princess Margaret Cancer Center, University Health Network, Toronto, ON M5G 2C4, Canada

**Keywords:** prostate cancer, anti-CTLA4, radiotherapy, radiorecurrence, brachytherapy

## Abstract

**Simple Summary:**

Advanced prostate cancer (PCa) is a prominent contributor to cancer-related fatalities and is associated with significant morbidity and mortality. Currently, addressing the local recurrence of the disease after radiation therapy (RT) poses a significant clinical challenge. We established the first syngeneic model of radiorecurrent PCa to evaluate the effectiveness of immune checkpoint inhibitors (ICIs) in combination with high-dose ionizing radiation (IR). We observed an enhanced anti-tumor response, which led to a delay in tumor growth and, in some cases, a complete elimination of tumors when combining IR with anti-CTLA4. This improvement was linked to an enhanced activation of total T cells, CD4+ helper T cells, and CD8+ cytotoxic T cells in both the draining lymph node and tumor. These results hold substantial potential for translation into clinical practice, serving as a proof of concept for the application of brachytherapy and immune checkpoint inhibitors (ICIs) in cases of recurrent disease.

**Abstract:**

High-risk prostate cancer (PCa) is a leading cause in cancer death and can elicit significant morbidity and mortality. Currently, the salvage of local disease recurrence after radiation therapy (RT) is a major clinical problem. Immune checkpoint inhibitors (ICIs), which enhance immune activation, have demonstrated clinical therapeutic promise in combination with ionizing radiation (IR) in certain advanced cancers. We generated the TRAMP-C2 HF radiorecurrent syngeneic mouse model to evaluate the therapeutic efficacy of ICIs in combination with RT. The administration of anti-PDL1 and/or anti-CTLA4 did not achieve a significant tumor growth delay compared to the control. The combination of IR and anti-PDL1 did not yield additional a growth delay compared to IR and the isotype control. Strikingly, a significant tumor growth delay and complete cure in one-third of the mice were seen with the combination of IR and anti-CTLA4. Immune cells in tumor-draining lymph nodes and tumor-infiltrating lymphocytes from mice treated with IR and anti-CTLA4 demonstrated an upregulation of genes in T-cell functions and enrichment in both CD4+ and CD8+ T-cell populations compared to mice given IR and the isotype control. Taken together, these results indicate enhancement of T-cell response in radiorecurrent PCa by IR and anti-CTLA4.

## 1. Introduction

In North America, one in eight men will be diagnosed with prostate cancer (PCa) in their lifetime and approximately 40,000 men will succumb to their disease yearly, making it a leading cause of cancer death [1,2]. In particular, men with high-risk PCa are at a significant risk of dying of their cancer, with an estimated eight-fold increase in PCa mortality rate compared to low-risk patients [3]. High-risk PCa is defined as locally advanced disease with no evidence of distant metastasis, a Gleason score of 8, or a prostate-specific antigen (PSA) value greater than 20.0 ng/mL [4].

High-risk PCa patients are at considerable risk of experiencing a local recurrence post radiotherapy, also termed radiorecurrent cancer [5]. The local recurrence of cancer can result in significant morbidity due to local invasion and reduce patient survival [6]. Additionally, high-risk PCa has a high propensity for subsequent metastases development and death [7,8,9]. Despite newer systemic agents (notably androgen receptor antagonists) that demonstrate improved survival for metastatic PCa patients, this remains an incurable situation [10,11]. For patients who have been previously treated with radiotherapy, tissue fibrosis can make salvage prostatectomy challenging to perform and is associated with significant morbidity [12]. Re-irradiation for focal salvage using high-dose-rate brachytherapy (HDR BT) is a management option; however, the relapse rate remains considerable and thus a more effective treatment regimen is needed to salvage local recurrence following radiotherapy [13,14,15].

Immunotherapy, especially immune checkpoint inhibitors (ICIs), are an emerging treatment that has quickly gained adoption in cancer therapy [16]. Most prevalent targets of ICIs include surface immune inhibitory molecules such as Cytotoxic T-Lymphocyte-Associated Protein 4 (CTLA4) and Program Cell Death Protein 1 (PD1), expressed by activated T cells, as well as the ligand of PD1, Program Cell Death Ligand 1 (PDL1). The binding of CTLA4 and PD1 to their respective ligands—CD80/CD86 and PDL1/PDL2 (program cell death ligand 2)—results in T-cell suppression [17]. ICIs can enable T-cell activation by blocking the interaction between checkpoint proteins and their ligands. Anti-CTLA4, anti-PD1, and anti-PDL1 therapy have achieved robust success across multiple cancer types, including melanoma, non-small-cell lung cancer (NSCLC), and colorectal cancer [16].

The use of ICIs has been actively evaluated by clinical trials for advanced PCa, with limited success overall [18,19]. Phase III clinical trials using anti-PD1/PDL1 and/or anti-CTLA4 have not yet achieved a significant improvement in overall survival (OS) in patients with metastatic castration-resistant PCa (mCRPC) [18,19,20]. The poor response of PCa to ICIs is unsurprising as it is characteristically an immunologically cold tumor. As such, with limited tumor infiltration and low tumor mutational burden (TMB), this diminishes neoantigen presentation and the subsequent anti-tumor immune response [18]. Interestingly, a higher instance of clinical response was observed in a small subset of patients with mismatch repair-deficient (dMMR) cells, a high TMB, or aberrations in homologous recombination repair (HRR) [21,22]. Currently, pembrolizumab (anti-PD1) is the only FDA-approved ICI for PCa patients with microsatellite instability-high (MSI-H) or dMMR cancer [23]. Radiotherapy may favorably modulate the immune tumor microenvironment to improve tumor response [24]. Indeed, the addition of ipilimumab in mCRPC following radiotherapy to bone metastases resulted in improved OS compared to the placebo [25].

Investigations into the use of ICIs have primarily been conducted in the context of mCRPC. The potential of ICIs as a therapy for locally radiorecurrent disease remains to be elucidated. Furthermore, the combination of ICIs with HDR BT may offer a new avenue for salvage treatment for local recurrence. HDR BT can induce significant tumor death to enhance the release of neoantigens for priming the anti-tumor T-cell response, complemented with T-cell activation by ICIs [26].

Here, we established, to our knowledge, the first syngeneic model of radiorecurrent PCa to assess the efficacy of ICIs alone and in combination with high-dose ionizing radiation (IR) as salvage interventions. ICIs as a monotherapy had no impact on tumor control. We found improved an anti-tumor response consisting of a significantly prolonged tumor growth delay, and in some instances, a durable complete resolution of tumors, with the combination treatment of IR and anti-CTLA4. This was attributed to the enhancement of T-cell activation in both tumor and draining lymph nodes. Together, our findings provide a promising avenue for the use of anti-CTLA4 to potentiate salvage radiotherapy in radiorecurrent PCa.

## 2. Materials and Methods

### 2.1. Generation of Radiation-Resistant Cell Line and Validation by Clonogenic Assay

TRAMP-C2 mouse prostate adenocarcinoma cells (Cat# CRL-2731, RRID:CVCL_3615, ATCC, Manassas, VA, USA) were mock-irradiated or irradiated by hypofractionation (10 Gy per fraction for five fractions) to generate parental (PAR) or radiation-resistant (HF) cells using a Faxitron X-ray Irradiator (Faxitron, Tucson, AZ, USA). TRAMP-C2 PAR and HF cells were seeded at 250, 500, 2000, and 4000 cells per well in triplicate onto a six-well plate and mock-irradiated (0 Gy) or irradiated with 4, 6, and 8 Gy doses of radiation, respectively. Then, cells were placed in a humidified CO_2_ incubator at 37 °C to allow colonies to form. Five days later, colonies were stained with crystal violet and counted. Survival was expressed as the relative plating efficiency of HF cells compared with that of the PAR cells.

### 2.2. Cell Cycle Assay

Cells were seeded at a density of 2 × 10^5^ cells per six-well plate and mock-irradiated or irradiated with 10 Gy by a Faxitron X-ray Irradiator. Cells were trypsinized, washed with PBS and fixed in ice-cold 80% ethanol in Hank’s Buffered Salt Solution (HBSS) (137 mM NaCl, 5.4 mM KCl, 0.25 mM Na_2_HPO_4_, 0.44 mM KH_2_PO_4_, 1.3 mM CaCl_2_, 1.0 mM MgSO_4_, 4.2 mM NaHCO_3_) for 30 min on ice. Fixed cells were collected by centrifugation, washed twice with PBS, and resuspended in 50 μg/mL propidium iodide (Sigma-Aldrich, Oakville, Ontario, Canada) with 0.6% NP-40 (Thermo Fisher Scientific, Waltham, MA, USA) and 0.1 mg/mL RNAse A in HBSS for 30 min at room temperature in the dark. Flow cytometry was performed using a FACS Calibur flow cytometer (BD Biosciences, Mississauga, ON, Canada). The cell cycle profile was analyzed using FlowJo 10.0.4 (RRID:SCR_008520, FlowJo LLC, Ashland, OR, USA).

### 2.3. β-Galactosidase Senescence Assay

Cells were seeded in a six-well plate (0 Gy at 5 × 10^4^ cells per well and 10 Gy at 1 × 10^5^ cells per well) and irradiated 24 h later. Cells were fixed and stained (4 days after mock irradiation or irradiation) for senescence-associated (SA)-β-galactosidase using the Senescence β-Galactosidase Staining Kit (Cell Signaling Technology, Whitby, ON, Canada) as per the manufacturer’s instructions. 

### 2.4. γH2AX Assay

Cells were seeded in a six-well plate (0 Gy at 5 × 10^4^ cells per well and 10 Gy at 1 × 10^5^ cells per well). The following day, cells were mock-irradiated or irradiated at 6 Gy and fixed for 5 min, 6 h and 24 h after radiation in ice-cold 80% ethanol. Post fixation, cells were stained using anti-phospho-histone H2A.X (Ser139) (MilliporeSigma, Burlington, MA, USA) and measured for flow cytometry analysis using a FACS Calibur flow cytometer (BD Biosciences, Mississauga, ON, Canada).

### 2.5. Proliferation Assay

Cells were seeded in triplicate (0.5 × 10^5^ cells/well for mock radiation, and 1.0 × 10^5^ for 6 Gy and 10 Gy radiation) in six-well plates and mock-irradiated or irradiated with 6 Gy or 10 Gy of radiation. Four days later, cells were trypsinized and stained with trypan blue, and the total viable cell number was determined using the Countess (Thermo Fisher Scientific, Waltham, MA, USA) automated cell counter.

### 2.6. Wound Healing Assay

Cells were plated onto six-well culture plates and grown to 100% confluency. A wound was made on the cell monolayers by a micropipette tip and images were taken 0, 7, and 10 h after the wound was made using Olympus CKX53 Microscope (Thermo Fisher Scientific, Waltham, MA, USA). Images were processed on the Fiji platform (NIH, Bethesda, MD, USA) for the calculation of wound area.

### 2.7. Soft Agar

Cells were resuspended in 0.25% (*w*/*v*) agar, plated in triplicate on a base layer of 0.4% agar in 24-well plates (700 cells/well) and placed in a humidified CO_2_ incubator at 37 °C. Twenty-five days later, colonies were counted.

### 2.8. In Vivo Therapy Experiments

Animal procedures were performed according to ARRIVE guidelines, with approval from Sunnybrook Research Institute Animal Care Committee (AUP#19689, approved 15 March 2019). Naive male 6–8-week-old immune-competent mice (C57BL/6) (Charles River Laboratories, Garfield Heights, OH, USA) were housed in groups of six (to limit fighting) in a pathogen-free facility with 12 h light cycles, in individually ventilated cages on woodchip bedding, with access to water and food ad libitum, at 22 °C. We conducted the daily monitoring of animals for lethargy, hunched or abnormal posture, failure to groom, weight loss of 20%, persistent self-trauma, ulcerated tumor, lack of grooming (fur) or ambulation. Mice were transplanted subcutaneously in the hindleg with either TRAMP-C2 PAR or HF cells (1 × 10^6^ cells). Tumor growth was monitored by caliper measurements. Treatment initiation commenced once tumors reached a palpable size of 70 mm^3^ (day 0) and randomly assigned to treatment groups. For IR-only treatments, radiation was delivered while under isoflurane using a Faxitron X-ray Irradiator (Faxitron, Tucson, AZ, USA) on day 0. Customized lead shielding was used to ensure radiation was delivered only to the tumor. For ICI-only treatments, mice were administered three cycles of either anti-PD-L1 (Genentech, San Francisco, CA, USA), anti-PD-L2 (Bio X Cell, Lebanon, NH, USA) or anti-CTLA4 (Bio X Cell, Lebanon, NH, USA), or their respective isotype controls (Bio X Cell, Lebanon, NH, USA) on days 0, 3, and 6. For IR + ICI treatments, radiation was delivered on day 0, followed by three cycles of ICIs on days 1, 4, and 7. When tumor volumes reached the endpoint of 500 mm^3^, mice were sacrificed. For the collection of tumor and organ tissues for downstream analysis, mice were sacrificed 24 h after the second cycle of ICI (day 4 for ICI-only and day 5 for IR + ICI). After each tumor was excised, half of the tumor was flash-frozen in liquid nitrogen and used for RNA extraction using the RNeasy Kit (Qiagen, Hilden, Germany). The other half of the tumor was enzymatically digested using the MACS Tumor Dissociation Kit (Miltenyi Biotec, Gaithersburg, MD, USA). Tumor-draining lymph nodes and spleens were excised and dissociated physically on a 40 µm cell strainer (Sarstedt Inc, Nümbrecht, Germany) into a single-cell suspension. The treatment sample size is based upon our previous experience, to provide a statistical power of at least 80% to detect a two-fold difference in mean tumor volume between groups, a tumor volume variation of up to 50% within groups, and a statistical significance level of alpha = 0.05. Mice that developed tumor ulceration were excluded from the study. Due to constraints in the experimental procedure, experimenters could not be blinded. 

### 2.9. NanoString Analysis 

Total RNA was quantified by Nanodrop (Thermo Fisher Scientific, Waltham, MA, USA), and RNA integrity was evaluated by a bioanalyzer. NanoString analysis was performed using the Mouse PanCancer Immune Profiling Panel on the nCounter Pro Analysis System (NanoString Technologies, Seattle, WA, USA). Gene expression results were analyzed and visualized using the R statistical environment (version 4.1.3 (3 October 2022); https://www.R-project.org/, accessed on 20 August 2023). All statistical analyses were conducted with packages from Bioconductor (https://www.bioconductor.org, RRID:SCR_006442, accessed on 20 August 2023) and CRAN (https://cran.r-project.org/web/packages/, accessed on 20 August 2023). NanoString data were imported and checked for quality using NanoStringQCPro v1.26.0 and RUVSeq v1.28.0. Differential expression analysis was conducted using DESeq2 v1.34.0 (RRID:SCR_000154). For tumor expression analyses, due to decreased sample integrity, raw *p*-values were plotted for the volcano plot and heatmaps. For lymph node samples, *p*-values were adjusted by the Benjamini–Hochberg method. Enrichment analysis (enricher) was conducted using ClusterProfiler v4.2.2 (RRID:SCR_016884), with msigdbr v7.5.1 gene sets and custom immune panel pathway annotations from NanoString Technologies. In lymph node samples, genes with a significant *p*-adj value were taken for pathway enrichment or depletion analysis. In tumor samples, all genes that had a fold-change of >0.5 were taken for enrichment analysis (similarly, genes with fold-change of <−0.5 were used for depleted pathways). The immune cell-type score was calculated using nSolver Advanced Analysis (NanoString Technologies, Seattle, WA, USA).

### 2.10. Flow Cytometry

Single cells from the tumor digestion were treated with Red Blood Cell Lysing Buffer Hybri-Max (Sigma-Aldrich, St. Louis, MO, USA). Immune cell subset distribution and activation was assessed by flow cytometry using a set panel of antibodies against cell surface markers: FITC anti-mouse CD45, APC-Cy7 anti-mouse CD3, PerCP/Cy5.5 anti-mouse CD4, PE-Cy7 anti-mouse CD8a, and PE anti-mouse FoxP3 (BioLegend, San Diego, CA, USA). The staining conditions were recommended by the manufacturer. Flow cytometry was performed using FACSymphony A5 (BD, Franklin Lake, NJ, USA). Flow data were analyzed using FlowJo (BD, Franklin Lake, NJ, USA). 

### 2.11. Ethics

The animal work was approved by the Animal Care Committee of Sunnybrook Research Institute, Toronto (Animal Use Protocol Number: 19689).

### 2.12. Statistics 

Statistical analysis was performed with GraphPad Prism Software Version 9.02 (GraphPad Software, San Diego, CA, USA). Statistical significance was assessed using the Student’s *t*-test. For Kaplan–Meier curves, statistical significance was assessed by the log-rank (Mantel–Cox) test. Statistical significance was given as **** *p*-value < 0.0001 *** *p*-value < 0.001, ** *p*-value < 0.01 and * *p*-value < 0.05; NS (not significant).

### 2.13. Role of Funding Source

The funding institutions were not involved in this study’s design, data collection, analysis, interpretation, or reporting.

## 3. Results

### 3.1. Generation and In Vitro Characterization of Radiorecurrent TRAMP-C2 HF

**Figure 1:**
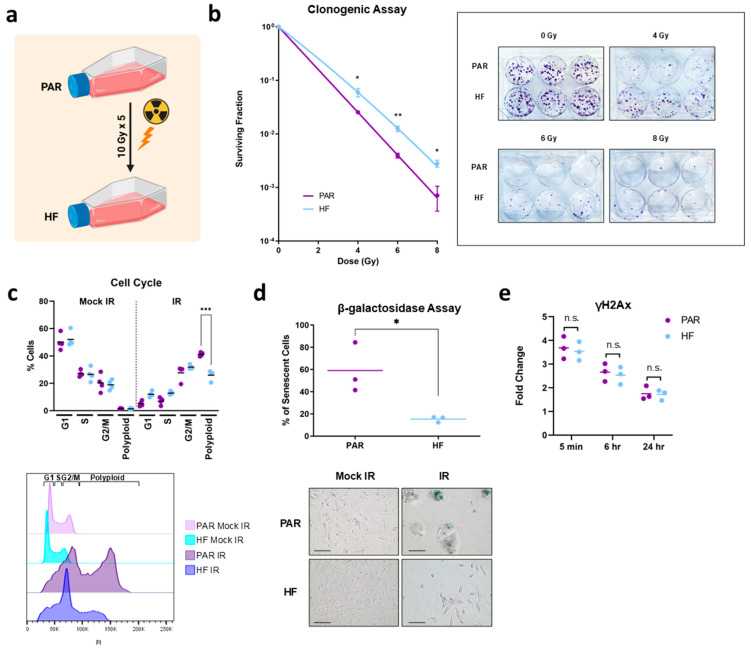
Generation and in vitro characterization of radiation-resistant TRAMP-C2 HF. (**a**) Schematic for generation of radiation-resistant TRAMPC2 HF from TRAMP-C2 PAR. Created with BioRender.com. (**b**) Clonogenic survival of TRAMP-C2 PAR compared to HF, surviving fraction was fitted on the linear quadratic model and represented on a logarithmic scale. Representative images of colonies treated with mock IR (0 Gy), 4 Gy, 6 Gy, and 8 Gy are shown. (**c**) Cell cycle profile of TRAMP-C2 PAR and HF cells 24 h post mock-IR (0 Gy) or IR (10 Gy). (**d**) β-galactosidase assay performed with TRAMP-C2 PAR and HF 4 days post mock IR (0 Gy) or 10 Gy. Representative images are shown (scale bar = 100 μm), senescent cells are stained blue. (**e**) γH2Ax foci analysis of TRAMP-C2 PAR and HF 5 min, 6 h and 24 h post IR (6 Gy) normalized to mock IR (0 Gy). Statistical significance is denoted; n.s. (not significant) *p* ≥ 0.05; * *p* < 0.05; ** *p* < 0.01; *** *p* < 0.001.

To establish a model of radiorecurrent PCa, we treated TRAMP-C2 PAR cells with 10 Gy per fraction for five fractions (mimicking a clinical prostate hypofractionated stereotactic radiotherapy schedule) to generate TRAMP-C2 HF cells (Figure 1a). TRAMP-C2 HF cells demonstrated increased radiation resistance compared to PAR cells (4 Gy *p* = 0.03 6 Gy *p* = 0.005, 8 Gy *p* = 0.02) (Figure 1b). PAR and HF cells displayed nearly identical cell cycle profiles in the G1, S and G2/M phases when treated with mock IR compared to IR (Figure 1c). This is consistent with the non-significant differences exhibited in cellular proliferation between mock IR and IR treatments (Figure S1a). Post IR treatment, however, a notable reduction in polyploid cells (*p* = 0.0001) was observed in HF cells compared to PAR cells (Figure 1c). Furthermore, a higher percentage of PAR cells post IR became enlarged with enhanced senescence-associated β-galactosidase activity (*p* = 0.03) compared to HF cells (Figure 1d). Taken together, this indicated that radiation resistance was attributed to fewer HF cells undergoing senescence post IR relative to PAR cells. No significant difference in the formation and resolution of γ-H2AX after IR was observed between PAR and HF cells (Figure 1e), which signified that DNA double-stranded repair was not altered in the HF cells. Additionally, there were no significant differences in migration found using the wound healing assay (Figure S1b). Overall, the radiation-resistant phenotype of the radiorecurrent TRAMP-C2 HF model was validated by in vitro characterization and compared to PAR cells.

### 3.2. Enhanced Tumorgenicity and Radiorecurrent of TRAMP-C2 HF Compared to PAR In Vivo

**Figure 2:**
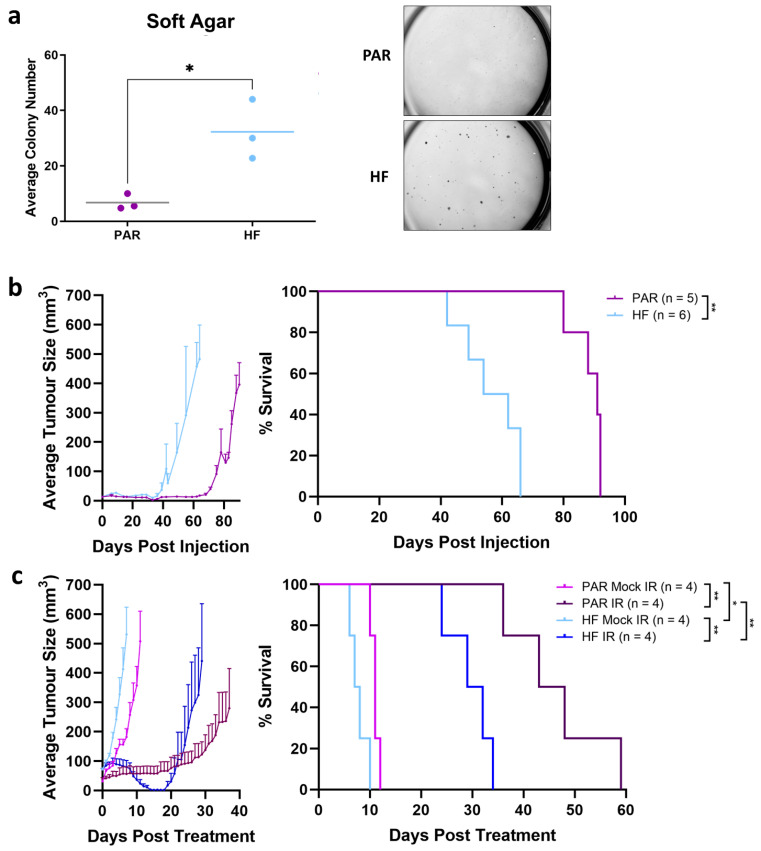
Enhanced tumorgenicity and radiation resistance of TRAMP-C2 HF compared to PAR. (**a**) Soft agar assay of TRAMP-C2 PAR and HF. Representative images of colony growth are shown. (**b**) Tumor growth kinetics and Kaplan–Meier survival of TRAMP-C2 PAR and HF tumors in C57BL/6 mice. (**c**) Tumor growth kinetics and Kaplan–Meier survival of TRAMP-C2 PAR and HF tumors treated with mock IR and 16 Gy. Statistical significance is denoted; * *p* < 0.05; ** *p* < 0.01.

We evaluated anchorage independent growth, which is an in vitro surrogate for tumorigenicity. TRAMP-C2 PAR and HF cells were seeded in soft agar (Figure 2a). After 25 days, HF cells formed larger and more numerous three-dimensional colonies than PAR cells (*p* = 0.02), indicating that HF cells have greater tumorigenic potential than PAR cells. We next sought to establish tumor xenografts using our PAR and HF cells to model radiorecurrent PCa in vivo. PAR and HF cells were injected subcutaneously into the hindleg of male immuno-competent C57BL/6 mice (Figure 2b). In accordance with observations in soft agar, the formation and time to endpoint (500 mm^3^) of HF tumors were notably quicker than PAR tumors (*p* = 0.002, median time to endpoint: PAR 91 days, HF 58 days). To assess for the radiation-resistant phenotype in vivo, PAR and HF subcutaneous tumors were administered mock IR or IR (16 Gy) after reaching a palpable size (70 mm^3^) (Figure 2c). While tumor growth delay was observed in both PAR (*p* = 0.007, median time to endpoint: PAR mock IR 11 days, PAR IR 45.5 days) and HF (*p* = 0.007, median time to endpoint: HF mock IR 7.5 days, HF IR 30.5 days) post IR, the growth delay in HF tumors was significantly shorter than in PAR tumors (*p* = 0.007). Interestingly, HF tumors also reached the endpoint significantly quicker than PAR tumors following mock IR (*p* = 0.02). In our syngeneic mouse model, the TRAMP-C2 HF tumors displayed radiation resistance and exhibited a more aggressive phenotype, similar to the radiorecurrent prostate cancer seen clinically [27]. 

### 3.3. Tumor Growth Delay Not Observed by Treatment by ICIs Alone

**Figure 3:**
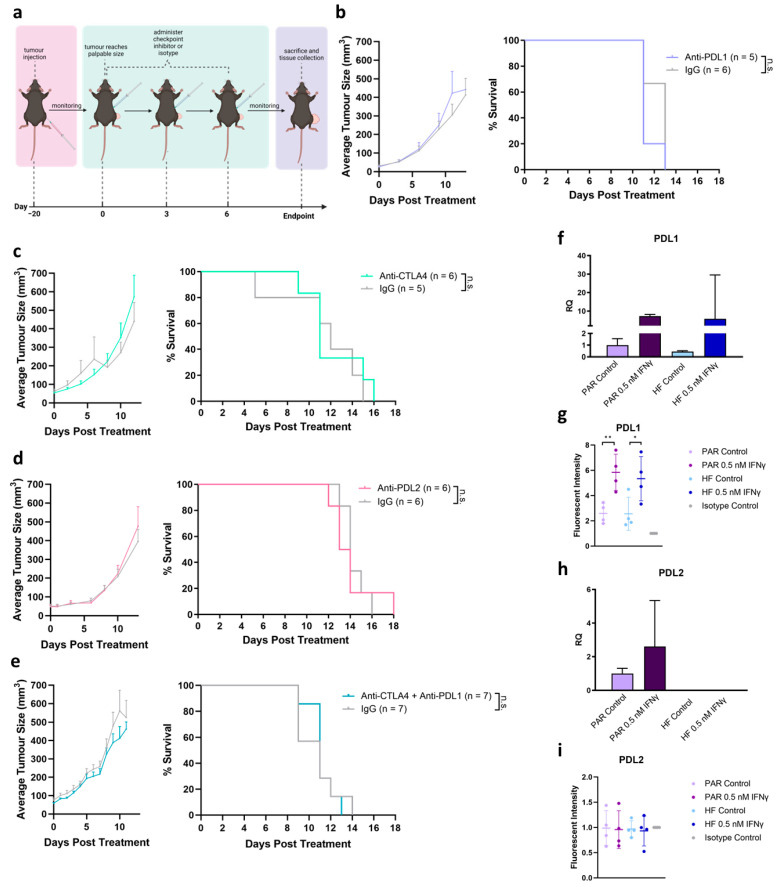
Tumor growth delay not observed by treatment by ICIs alone. (**a**) Schematics of C57BL/6 mice bearing TRAMP-C2 HF tumors treated with ICIs. Created with BioRender.com. (**b**–**e**) Tumor growth kinetics and Kaplan–Meier survival of TRAMP-C2 HF tumors treated with (**b**) anti-PDL1, (**c**) anti-CTLA4, (**d**) anti-PDL2, or (**d**) combination of anti-CTLA4 and anti-PDL1 compared to their respective IgG isotype controls. (**f**) qPCR of PDL1 expression in TRAMP-C2 PAR and HF cells post 24 h treatment with 0.5 nM IFNγ or vehicle control. (**g**) Flow cytometry analysis of PDL1 expression by fluorescent intensity normalized to isotype control in TRAMP-C2 PAR and HF cells post 24 h treatment with 0.5 nM IFNγ or vehicle control. (**h**) qPCR of PDL2 expression in TRAMP-C2 PAR and HF cells post 24 h treatment with 0.5 nM IFNγ or vehicle control. (**i**) Flow cytometry analysis of PDL2 expression by fluorescent intensity normalized to isotype control in TRAMP-C2 PAR and HF cells post 24 h treatment with 0.5 nM IFNγ or vehicle control. Statistical significance is denoted; n.s. (not significant) *p* ≥ 0.05; * *p* < 0.05; ** *p* < 0.01.

To evaluate the anti-tumor effect of immune checkpoint inhibitors on radiorecurrent PCa, C57BL/6 mice were inoculated with TRAMP-C2 HF tumors. Once the tumors reached a palpable size, mice were administered checkpoint inhibitors on days 0, 3, and 6 (Figure 3a). Single-agent treatment with anti-PDL1 did not improve the tumor growth delay compared to the IgG isotype control (median time to endpoint: IgG 13 days, anti-PDL1 11 days; Figure 3b). Concurrently, tumors were either excised after two cycles of IgG/anti-PDL1 or at the endpoint for the flow cytometry analysis of tumor infiltration (Figure S2a,b). No notable change in CD45+ cells, total T cells, T helper cells, or T cytotoxic cells were detected between treatments (IgG vs. anti-PDL1) or timepoints (during treatment vs. endpoint) (Figure S2a,b). Evidently, anti-PDL1 was not sufficient in promoting T-cell infiltration into the tumor when administered alone. Similarly, the administration of single-agent anti-CTLA4 (median time to endpoint: IgG 12 days, anti-CTLA4 11 days; Figure 3c) and anti-PDL2 (median time to endpoint: IgG 14 days, anti-PDL2 13.5 days; Figure 3d) did not achieve significant a tumor growth delay compared to their respective isotype controls. Furthermore, treatment using a combination of anti-PDL1 and anti-CTLA4 did not improve tumor control (Figure 3e). TRAMP-C2 PAR and HF cells express PDL1 RNA and protein at baseline and significantly upregulate PDL1 following treatment with the pro-inflammatory cytokine, IFNγ (Figure 3f,g). However, PDL2 RNA was only detectable in PAR cells, and protein was undetectable in both PAR and HF, irrespective of stimulation (Figure 3h,i).

### 3.4. Tumor Response Induced by Combination of Radiation and Anti-CTLA4

**Figure 4:**
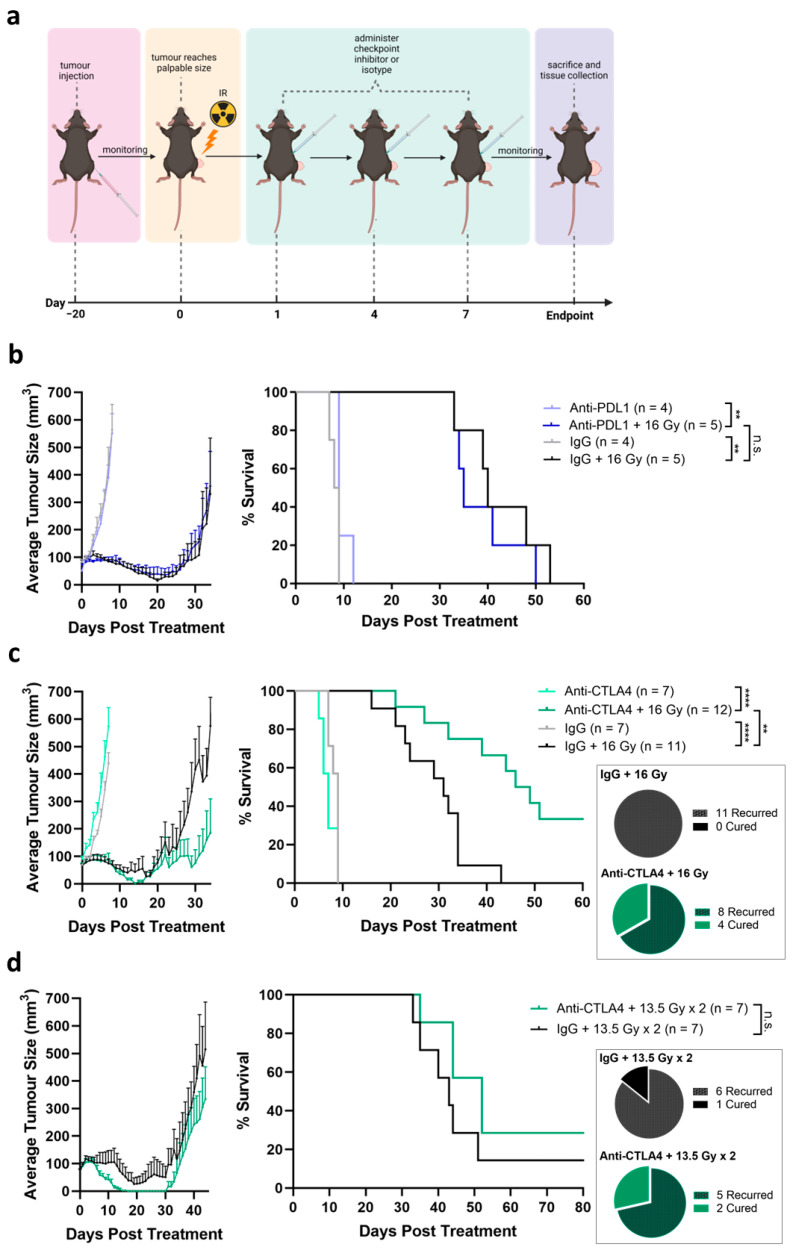
Tumor response induced by combination of radiation and antiCTLA4. (**a**) Schematics of C57BL/6 mice bearing TRAMP-C2 HF tumors treated with combination of single IR fraction (16 Gy) and ICIs. Created with BioRender.com. (**b**–**d**) Tumor growth kinetics and Kaplan–Meier survival of TRAMP-C2 HF tumors treated with (**b**) 16 Gy in combination with anti-PDL1 or IgG isotype control and (**c**) 16 Gy in combination with anti-CTLA4 or IgG isotype control. (**d**) Two fractions of 13.5 Gy in combination with anti-CTLA4 or IgG isotype. Statistical significance is denoted; n.s. (not significant) *p* ≥ 0.05; ** *p* < 0.01; **** *p* < 0.0001.

It is hypothesized that high-dose radiation can induce significant tumor cell death and the release of neoantigens to prime the anti-tumor immune response, which can be further enhanced by ICIs. To assess this hypothesis, tumors of a palpable size were given a single fraction of 16 Gy IR on day 0, followed by three doses of ICIs on days 1, 4, and 7 (Figure 4a). The dose of 16 Gy is similar to that of single-fraction salvage radiotherapy [28,29]. Compared to monotherapy anti-PDL1 or isotype IgG, the addition of 16 Gy IR resulted in observable tumor regression and a growth delay (anti-PDL1 *p* = 0.003, IgG *p* = 0.001; median time to endpoint anti-PDL1 8.5 days, 16 Gy + anti-PDL1 35 days, IgG 9 days, 16 Gy + IgG 40 days) (Figure 4b). However, the time to endpoint of the combination of anti-PDL1 and 16 Gy did not differ compared to the combination of isotype control and IR (Figure 4b). In stark contrast, a significant tumor growth delay was seen with the combination of IR and anti-CTLA4 (*p* < 0.0001; median days to endpoint: anti-CTLA4 6.5 days, 16 Gy + anti-CTLA4 47.5 days) compared to IR and isotype control (*p* < 0.0001; median days to endpoint: IgG 9 days, 16 Gy + IgG 31 days; Figure 4c). Strikingly, four mice showed complete tumor regression and remained tumor-free for at least 60 days (33.3% cure rate) following treatment initiation (Figure 4c). 

We next tested two-fraction radiotherapy, which is clinically used for salvage radiotherapy [15,28], in combination with checkpoint therapy. Mice were given one fraction of 13.5 Gy IR on day 0, followed by three doses of ICIs on days 1, 4, and 7. Additionally, a second fraction of 13.5 Gy was given on day 10, followed by three more doses of ICIs on days 11, 14, and 17. The combination of two fractions of IR with anti-CTLA4 yielded a favorable trend in tumor growth delay compared to two-fraction IR with IgG, although it was not statistically significant (median days to endpoint: 13.5 Gy × 2 + anti-CTLA4 44 days, 13.5 Gy × 2 + IgG 41.5 days; Figure 4d). IR treatment with two 13.5 Gy fractions with anti-CTLA4 resulted in a full tumor cure in two mice up to at least 80 days post treatment initiation (28.5% cure rate), similar to the cure rate observed following treatment with single-fraction 16 Gy with anti-CTLA4. Interestingly, one mouse treated with 13.5 Gy × 2 and IgG was also cured of its tumor (14.3% cure rate). Overall, two-fraction IR resulted in improved tumor control compared to single-fraction IR without anti-CTLA4, while two IR fractions did not result in further benefit in combination with anti-CTLA4 relative to the single fraction.

### 3.5. Treatment with 16 Gy + Anti-CTLA4 Promoted T-Cell Activation in TDLNs

**Figure 5:**
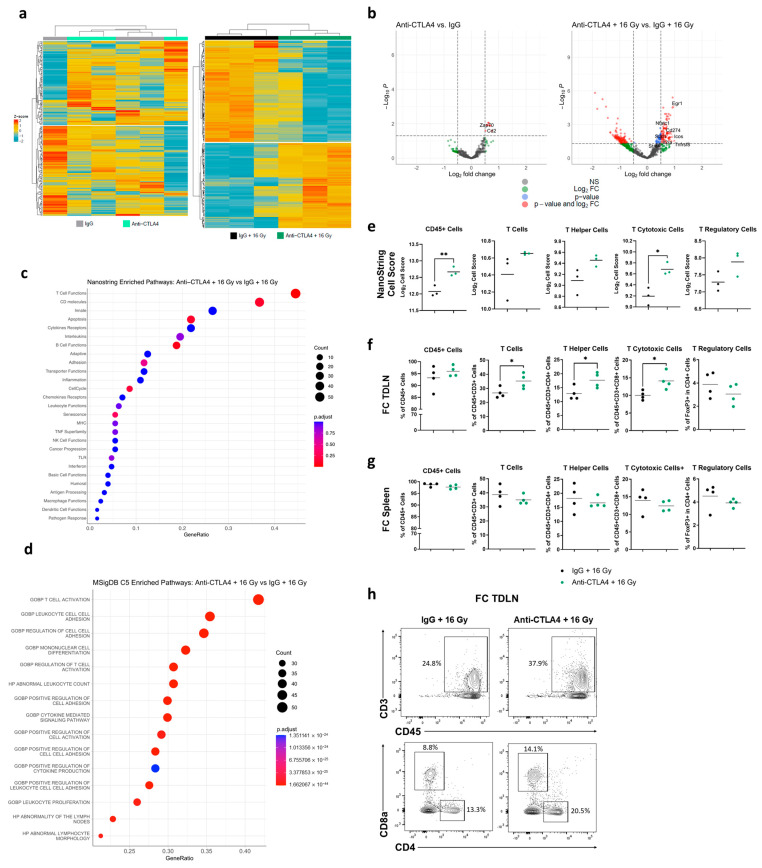
Treatment with 16 Gy + anti-CTLA4 promoted T-cell activation in TDLNs. (**a**) Heatmaps of differentially expressed immune-related genes of TDLNs from mice treated with anti-CTLA4 vs. IgG and 16 Gy + anti-CTLA4 vs. 16 Gy + IgG. (**b**) Volcano plots of differentially expressed immune-related genes of TDLNs from mice treated with anti-CTLA4 vs. IgG and 16 Gy + anti-CTLA4 vs. 16 Gy + IgG. Fold-change cut-off 0.5, adjusted *p*-values (Benjamini–Hochberg) <0.05. (**c**) Enrichment analysis using NanoString PanCancer Immune Profiling Panel for significant genes (by *p*-adj value) in 16 Gy + anti-CTLA4 group. (**d**) Enrichment analysis using Mouse MSigDB C5 for significant genes (by *p*-adj value) from NanoString PanCancer Immune Profiling Panel in 16 Gy + anti-CTLA4 group. (**e**) Cell score for CD45+ cells, total T cells, T helper cells, T cytotoxic cells, and T regulatory cells of TDLNs from mice treated with 16 Gy + anti-CTLA4 vs. 16 Gy + IgG. (**f**) Flow cytometry analysis of CD45+ cells, total T cells, T helper cells, T cytotoxic cells, and T regulatory cells of TDLNs from mice treated with 16 Gy + anti-CTLA4 vs. 16 Gy + IgG. (**g**) Flow cytometry analysis of CD45+ cells, total T cells, T helper cells, T cytotoxic cells, and T regulatory cells of spleens from mice treated with 16 Gy + anti-CTLA4 vs. 16 Gy + IgG. (**h**) Representative flow gating for total T cells, T helper cells, and T cytotoxic cells of TDLNs from mice treated with 16 Gy + anti-CTLA4 vs. 16 Gy + IgG. Statistical significance is denoted; * *p* < 0.05; ** *p* < 0.01.

To investigate the underlying immunological mechanism that drove the enhanced therapeutic effect of 16 Gy + anti-CTLA4, tumor-draining lymph nodes (TDLNs) and spleens were excised after two cycles of IgG/anti-CTLA4 or one fraction of 16 Gy IR and two cycles of IgG/anti-CTLA4 (Figure S3a). NanoString analysis demonstrated that TDLNs of mice treated with IgG and anti-CTLA4 alone did not show distinct immune-related gene expression (Figure 5a,b). Interestingly, TDLNs from mice that received 16 Gy + IgG revealed very distinct differential immune-related gene expression compared to those who received 16 Gy + anti-CTLA4 (Figure 5a,b). Using curated gene lists from the NanoString PanCancer Immune Profiling Panel, 16 Gy + anti-CTLA4 yielded the enrichment of genes in T-cell functions (Figure 5c), while no pathways of significance were found with the most depleted genes (Figure S3b). Consistent with NanoString analysis, pathway analysis using Mouse MSigDB found T-cell activation to be the most enriched pathway in the 16 Gy + anti-CTLA4 group (Figure 5d). Furthermore, upregulated genes in the combination treatment were associated with pathways involved in T-cell and leukocyte responses (Figure 5d and Figure S3c). Cell type-specific mRNAs were used to calculate the immune cell-type score using NanoString nSolver (Figure S3d). We found significant increases in cell-type scores for CD45+ cells and cytotoxic T cells, as well as an upward trend in total T cells, helper T cells, and regulatory T cells (Tregs), in the 16 Gy + anti-CTLA4 treatment compared to 16 Gy + IgG (Figure 5e). 

The flow cytometry analysis of 16 Gy + anti-CTLA4-treated TDLNs found a notable increase in total T cells, helper T cells, and cytotoxic T cells, and no significant changes in CD45+ cells and Tregs compared to the TDLNs given 16 Gy + IgG (Figure 5f,h). Overall, the gene expression and flow cytometry results provided evidence for enhanced T-cell activation and expansion in TDLNs of mice in the 16 Gy + anti-CTLA4 group. Additionally, no significant differences in immune cell populations were detected in spleens from the 16 Gy + IgG arm compared to the 16 Gy + anti-CTLA4 arm (Figure 5g), strongly suggesting that the observed enhanced T-cell response in TDLN is tumor specific. 

### 3.6. Treatment with 16 Gy + Anti-CTLA4 Promoted T-Cell Activation in Tumors

**Figure 6:**
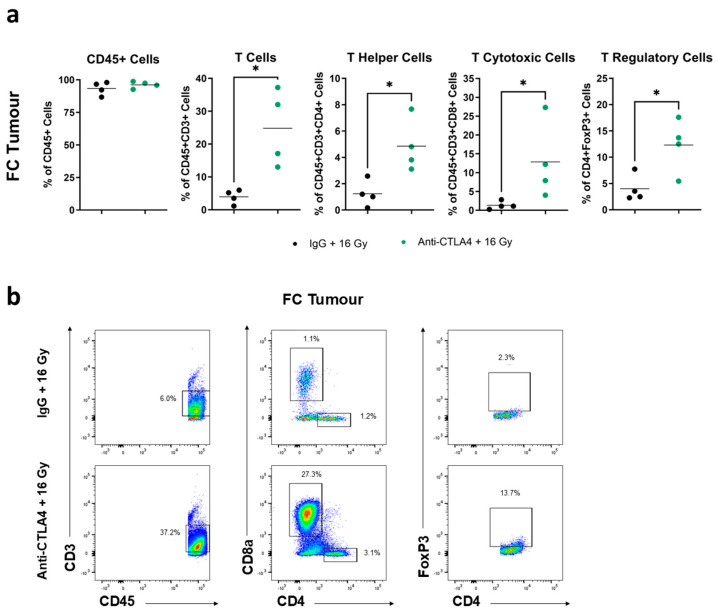
Treatment with 16 Gy + anti-CTLA4 promoted T-cell activation in tumors. (**a**) Flow cytometry analysis of CD45+ cells, total T cells, T helper cells, T cytotoxic cells, and T regulatory cells of tumors from mice treated with 16 Gy + anti-CTLA4 vs. 16 Gy + IgG. (**b**) Representative flow gating for total T cells, T helper cells, T cytotoxic cells, and Tregs of tumors from mice treated with 16 Gy + anti-CTLA4 vs. 16 Gy + IgG. Statistical significance is denoted; * *p* < 0.05.

In addition to TDLNs and spleens, corresponding tumors were excised from the same mice concurrently (Figure S3a). The NanoString analysis of tumors found that treatment groups that received IR (16 Gy + IgG and 16 Gy + anti-CTLA4) displayed significant differential gene expression compared to no-IR groups (IgG and anti-CTLA4) (Figure S4a,b). Pathway analysis using the NanoString PanCancer Immune Profiling Panel unveiled CD molecule-related genes to be most enriched in IR groups versus no-IR groups (Figure S4c). Closer inspection within no-IR groups showed that IgG and anti-CTLA4 alone did not induce a distinct pattern of differential gene expression. Similar to TDLNs, genes in the 16 Gy + IgG group displayed a trend for differential expression compared to the 16 Gy + anti-CTLA4 group, although this was not statistically significant (Figure S4d). Furthermore, the most-enriched immune-related genes in 16 Gy + anti-CTLA4-treated tumors are involved in CD molecules and T-cell function, again mirroring observations in TDLNs (Figure S4e,f). Flow cytometry analysis revealed an accumulation of tumor-infiltrating total T cells, T helper cells, T cytotoxic cells, and Tregs after treatment with 16 Gy + anti-CTLA4 relative to 16 Gy + IgG (Figure 6a,b). No notable changes in CD45+ cells were seen between the two treatments (Figure 6a). Overall, the evidence suggested an enhanced tumor infiltration by T cells following the combination of 16 Gy + anti-CTLA4.

## 4. Discussion

Local recurrence post-prostate RT is a significant clinical challenge characterized by aggressive disease and limited salvage interventions [27]. To our knowledge, TRAMP-C2 HF is the first syngeneic model for localized radiorecurrent PCa. To better recapitulate the acquisition of radiation resistance in the clinical setting, the HF cells were generated through treatment using dose fractionation synonymous to stereotactic body radiation therapy (SBRT), which is being rapidly adopted for PCa RT [30]. We found that the TRAMP-C2 HF model demonstrated increased radiation resistance, senescence, and anchorage independent growth compared to PAR in vitro. Furthermore, TRAMP-C2 HF also displayed increased radiation resistance and tumorgenicity in vivo. The overall aggressive phenotype of the TRAMP-C2 HF model captures that of radiorecurrent cancer seen in patients [5].

Single-agent anti-PDL1 did not prolong tumor survival compared to isotype IgG despite the detectable expression of PDL1 on TRAMP-C2 HF cells. As such, PDL1 expression alone was not prognostic for the response to anti-PDL1, which is consistent with results observed in PDL1+ vs. PDL1- mCRPC patients receiving pembrolizumab [21]. Furthermore, TRAMP-C2 HF tumors exhibited a low infiltration of T cells (<10%) both during treatment as well as at the endpoint. Low T-cell infiltration has previously been observed in the TRAMP tumor model [31]. This reflected the immunologically cold nature commonly seen with PCa and provided evidence for the lack of immune modulation exhibited by anti-PDL1 alone. Low tumor-infiltrating lymphocytes (TILs) have been shown to correlate with poor outcomes in patients [32]. In our models, PDL2 protein was undetectable, even under stimulatory conditions with IFNγ, potentially explaining the lack of efficacy of anti-PDL2. The absence of anti-tumor activity of anti-PDL2, anti-CTLA4 and combination of anti-PDL1 and anti-CTLA4 further demonstrated that ICIs alone were unable to induce a robust immune response. Similarly, no overall benefit was found with mCRPC patients treated with a combination of ipilimumab and nivolumab [22].

Emerging evidence indicates the importance of the immune system in governing RT response [33]. A NanoString analysis on tumor biopsies taken from patients with localized PCa receiving HDR BT caused a shift in the gene signature of immunologically cold tumors to a more inflammatory expression seen with hot tumors [26]. Both durvalumab and pembrolizumab post RT improved OS in NSCLC patients [34,35]. Therefore, IR combined with ICIs may prime the immune system to recognize and target recurrent cancer [33]. Here, we used a single fraction of 16 Gy to emulate HDR salvage brachytherapy [36]. Anti-PDL1 + 16 Gy provided no additional benefit relative to IgG + 16 Gy, whereas anti-CTLA4 + 16 Gy achieved a notable tumor growth delay and fully cured one-third of the mice. Interestingly, OS was improved in mCRPC patients that received radiotherapy to metastatic sites in the bone prior to the initiation of ipilimumab, compared to the placebo [25]. 

Tumor cell death by IR releases neoantigens that can be captured by antigen-presenting cells (APCs) [33]. Subsequently, there is APC traffic to lymph nodes, where the priming and expansion of T cells against tumor neoantigens take place [33,37]. Activated tumor-specific T cells then exit lymph nodes for tumor clearance [33,37]. T-cell priming and anti-tumor responses can be further enhanced with ICIs [38]. As such, the high tumor infiltration of T cells has demonstrated prognostic value for patient response [39], including for ICIs [32]. We postulate that the therapeutic response by 16 Gy + anti-CTLA4 was attributed to this mode of IR-induced anti-tumor immunity. Our results support this as elevated total CD3+ T cells were detected in both TDLNs and tumors, signifying enhanced T-cell activation and infiltration, respectively.

Notably, IgG with two fractions of 13.5 Gy was also able to cure a subset of mice, and the addition of anti-CTLA4 to two fractions of 13.5 Gy did not further improve tumor control. Therefore, the additional fraction allowed for enhanced tumor killing by IR compared to the single fraction. However, it did not further increase tumor immunogenicity as no additional benefit in combination with anti-CTLA4 was detected. In conjunction, two fractions of high-dose RT were much less well-tolerated than a single dose, as drastic skin toxicity was observed.

CTLA4 is expressed on activated T cells and has a higher affinity for binding with costimulatory molecule CD80/86 on antigen presenting cells (APCs) than the coactivation receptor CD28, allowing for T-cell inhibition [40]. Tregs constitutively express CTLA4 to promote T-cell suppression [40]. In contrast to CTLA4, the expression of PD1 can be found on activated T cells as well as other immune cell types such as B cells and myeloid cells [41]. Additionally, PD1 ligands also have a wider range of expression, with PDL1 found on immune cells, non-hematopoietic cells, and tumor cells, and PDL2 found primarily on dendritic cells and monocytes [41,42]. As such, inhibition by CTLA4 takes place during the priming stage, mainly in the TDLNs, as CD80/86 are transiently expressed on professional APCs [17]. In contrast, the distribution of PD1 and PDL1/2 across different tissues allows for suppression function during the effector stage in the peripheral tissues [17].

T helper cells have demonstrated essential roles in the priming of T cytotoxic cells in TDLNs [43]. We observed an expansion of CD4+ T helper and CD8+ T cytotoxic cell populations in TDLNs as well as enhanced tumor infiltration in the 16 Gy + anti-CTLA4 arm. The lack of benefit from 16 Gy + anti-PDL1 is likely attributed to insufficient T-cell expansion during the priming stage, which did not produce an effector response robust enough to be augmented by anti-PDL1. Therefore, in our recurrent model, enhanced T-cell expansion during the priming stage in TDLNs was required for an extended anti-tumor effect and was only achieved by the combination of 16 Gy + anti-CTLA4.

Our NanoString data further supported this as differential gene expression in TDLNs was seen between the 16 Gy + IgG arm and 16 Gy + anti-CTLA4 arm, with most enriched genes being involved in T-cell function. Notably, we observed upregulated genes in Th1 cell polarization (*Stat1/4*, *Egr1*, *Il2ra*) and T-cell activation (*Tnfrsf8*, *Icos*, *Cd274*, *Nfatc1*) [17,44,45,46,47,48,49,50]. A similar trend, albeit not significant, was seen in differential gene expression in tumors, with enrichment in T-cell function genes (*Pdcd1lg2*, *Pdcd1*, *Il7r*, *Il1b*, *Runx3*) [17,49,51,52,53]. Since the 16 Gy + anti-CTLA4 combination acts directly on priming and activating T cells in TDLNs, and subsequently indirectly on tumor infiltration, this is consistent with having more notable differential gene expression in TDLNs compared to tumors. Indeed, as tumors received direct IR treatment, significant differential gene expression was observed in tumors from irradiated arms vs. non-irradiated arms.

Tregs were scarcely found in TDLNs and tumors, and no significant alteration by 16 Gy + anti-CTLA4 was seen compared to the control in TDLNs. This signified that the expansion of T cells was not being negatively regulated by Tregs. The lack of Treg depletion provided evidence that anti-CTLA4 mainly prevented the receptor ligand binding of activated T cells as opposed to inducing the antibody-dependent cellular cytotoxicity of Tregs. The absence of Treg depletion following ipilimumab treatment had also been observed in PCa patients [54]. The low presence of tumor Tregs signified that immune suppression was attributed primarily to poor T-cell infiltration rather than inhibition by Tregs. Therefore, the increased Tregs in tumors from 16 Gy + anti-CTLA4 may be a negative feedback response to enhanced tumor infiltration by T cells.

Previous pre-clinical studies using the TRAMP model revealed the efficacy of anti-CTLA4 therapy following the administration of an irradiated cell-based cancer vaccine in radiation-naïve cancer through the activation of effector T-cell response [55,56,57]. Building on these findings, our results showcased that the treatment of tumors by high-dose radiotherapy alone effectively functions as in situ vaccination, which promoted the anti-tumor T-cell response in combination with anti-CTLA4. Furthermore, this pre-clinical model is unique in recapitulating locally advanced radiorecurrent disease, which was demonstrated both clinically and, in our model, to be more pathologically aggressive and treatment-resistant compared to radiation-naïve cancer.

While we aimed to establish a model that most accurately reflects the clinical scenario, there are limitations to this study. The established model is a subcutaneous murine model, which may not accurately capture all the underlying biology of human PCa. Future experiments should look to recapitulate results in orthotopic and/or humanized mouse models. The dose, fractionation, and sequencing of IR with ICIs have profound effects on treatment efficacy [58,59,60]. While we explored IR dosing and fractionation, we did not evaluate the sequencing of ICIs with IR. Future work should examine the use of neoadjuvant ICIs prior to IR, which have been shown to improve response. Androgen deprivation therapy (ADT) is commonly used in conjunction with radiotherapy and has been found to elicit immune stimulatory effects [61,62]. As such, further research should aim to evaluate any potential benefits from combining ADT with ICIs and IR. We also acknowledge that our correlative studies using NanoString and flow cytometry were restricted by a limited number of mice per condition (*n* = 4). The use of biomarkers including TMB, MSI-H, dMMR, and TILs have demonstrated limited prognostic or predictive value in the context of ICIs for PCa [18]. While 16 Gy + anti-CTLA4 yielded an overall tumor growth delay, only a cohort of mice were fully cured. Therefore, additional investigations for predictive biomarkers in responders versus non-responders will be required, which will identify potential targets for patient biomarker selection. 

## 5. Conclusions

Altogether, our results indicated a well-tolerated anti-tumor effect using high-dose radiation combined with anti-CTLA4 in radiation-recurrent PCa, with a one-third cure rate. This was achieved through the promotion of T-cell activation in TDLNs and infiltration back into the tumor. The findings of this pre-clinical study have significant translational promise as proof of principle for the use of brachytherapy and ICIs in recurrent disease. Further investigations of the optimization of treatment schedule and biomarker discovery will be necessary to allow for the implementation of this intervention strategy in early clinical trials.

## Data Availability

Images from this paper are not reprinted/have not been previously published. The data generated in this study are available upon request to the authors. Correspondence should be addressed to H.W. and S.K.L.

## Supplementary Materials

The following supporting information can be downloaded at: https://www.mdpi.com/article/10.3390/cancers16162839/s1, Figure S1. in vitro characterization of radiation-resistant TRAMP-C2 HF. Figure S2. Tumor growth delay not observed by treatment by ICIs alone. Figure S3. Treatment with 16 Gy + anti-CTLA4 promoted T-cell activation in TDLNs. Figure S4. Treatment with 16 Gy + anti-CTLA4 promoted T-cell activation in tumors.
