# Supplementary material for: Potentiating Salvage Radiotherapy in Radiorecurrent Prostate Cancer Through Anti-CTLA4 Therapy: Implications from a Syngeneic Model
Source: Cancers (Basel). 2024 Aug 14;16(16):2839. doi: 10.3390/cancers16162839 (PMC11352774; doi:10.3390/cancers16162839)
Supplement: Supplementary file 1 [file cancers-16-02839-s001.zip › cancers-3107391-supplementary.pdf]

Supplementary Materials: Potentiating Salvage Radiotherapy in Radiorecurrent Prostate Cancer through Anti-CTLA4 Therapy: Implications from a Syngeneic Model

Hanzhi Wang, Linsey Gong, Xiaoyong Huang, Stephanie D. White, Hans T. Chung, Danny Vesprini, Tera N. Petchiny, Emmanouil Fokas, Hansen He, Robert S. Kerbel and Stanley K. Liu

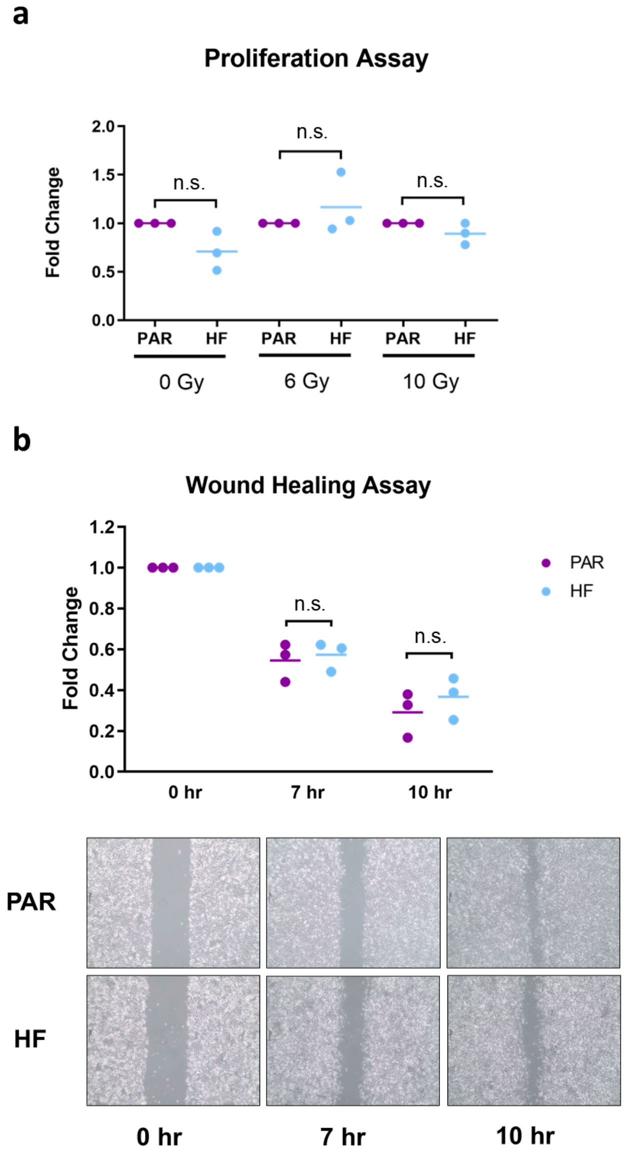

Supplementary Figure S1. In vitro characterization of radiation resistant TRAMP-C2 HF. (a) Proliferation assay of TRAMP-C2 PAR and HF cells 4 days post mock-IR (0 Gy), 6 Gy, or 10 Gy. (b) Wound healing assay of TRAMP-C2 PAR and HF 0 hr, 7 hr and 10 hr post wound infliction. Representative images are shown. Statistical significance is denoted; n.s. (not significant) $p \geq 0.05$.

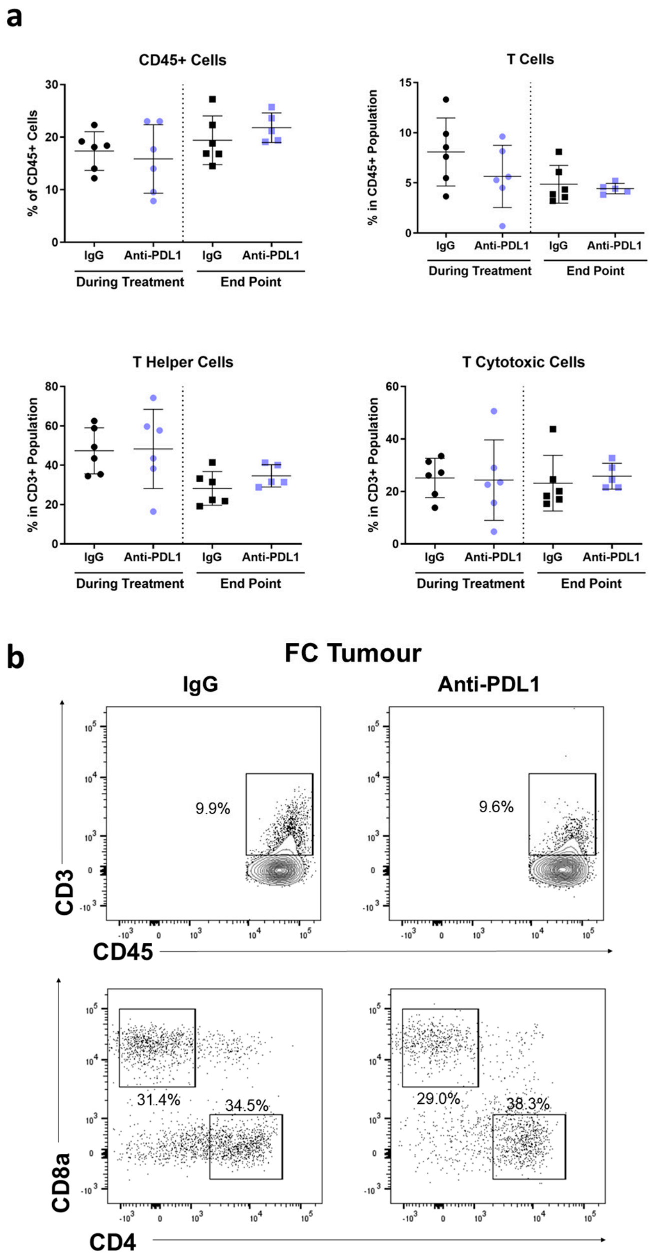

Supplementary Figure S2. Tumor growth delay not observed by treatment by ICIs alone. (a) Flow cytometry analysis of CD45+ cells, total T cells, T helper cells, and T cytotoxic cells of tumors from mice treated with anti-PDL1 vs. IgG during treatment and at endpoint. (b) Representative flow gating for total T cells, T helper cells, and T cytotoxic cells of tumors from mice treated with anti-PDL1 vs. IgG.

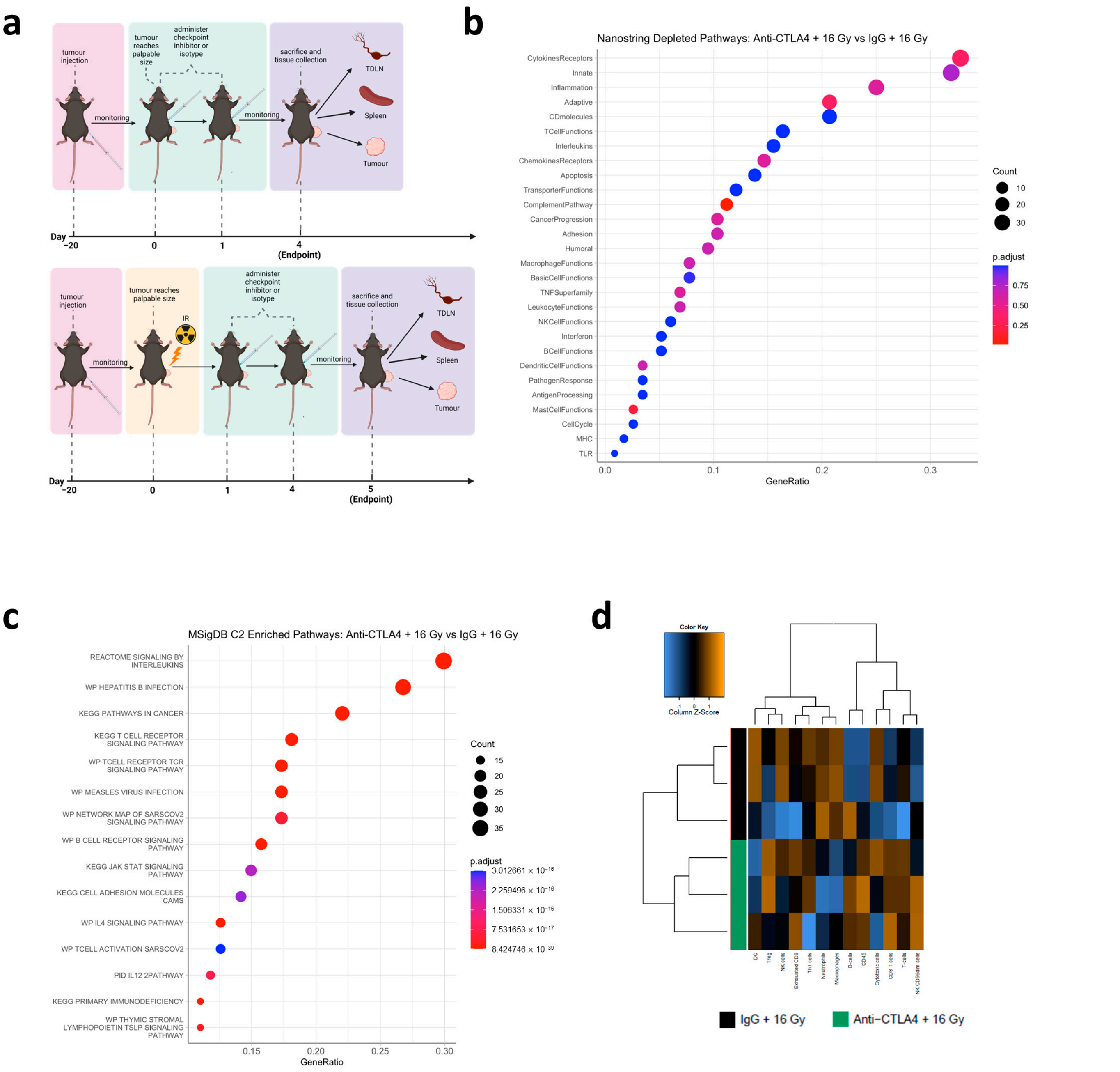

Supplementary Figure S3. Treatment with 16 Gy + anti-CTLA4 promoted T cell activation in TDLNs. (a) Schematics of tissue collection (TDLN, spleen, and tumor) from C57BL/6 mice bearing TRAMP-C2 HF tumors treated with anti-CTLA4/IgG alone or in combination with single IR fraction (16 Gy) after 2 cycles of anti-CTLA4/IgG. Created with BioRender.com. (b) Enrichment analysis using NanoString PanCancer Immune Profiling Panel for depleted genes in 16 Gy + anti-CTLA4 group. (c) Enrichment analysis using Mouse MSigDB C2 for significant genes (by p-adj value) from NanoString PanCancer Immune Profiling Panel in 16 Gy + anti-CTLA4 group. (d) Heatmap of immune cell type score of TDLNs treated with 16 Gy + anti-CTLA4 vs. 16 Gy + IgG.

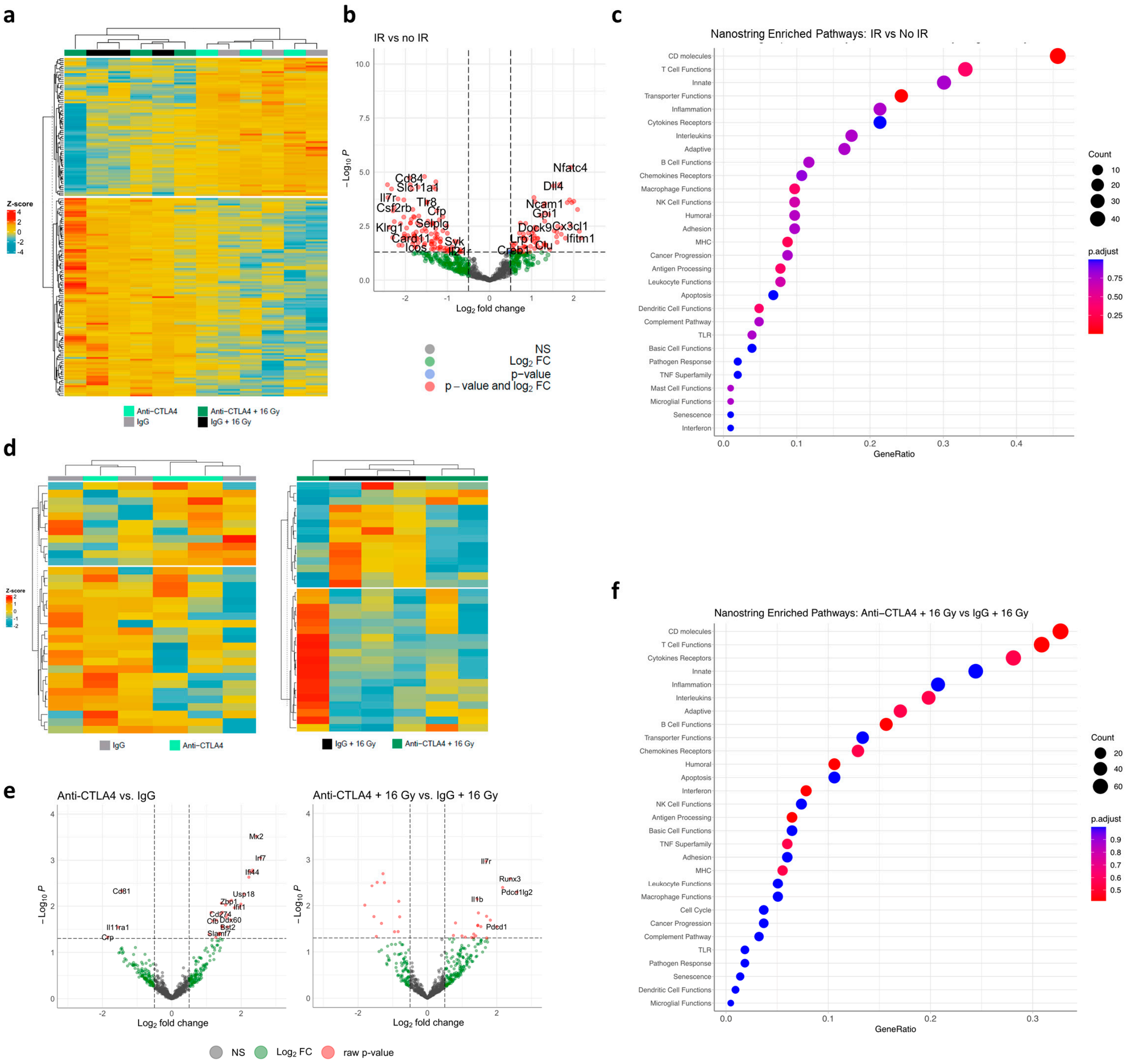

Supplementary Figure S4. Treatment with 16 Gy + anti-CTLA4 promoted T cell activation in tumors. (a) Heatmap of differentially expressed immune-related genes of tumors from mice treated with no IR (anti-CTLA4 and IgG) vs. with IR (16 Gy + anti-CTLA4 and 16 Gy + IgG). (b) Volcano plot of differentially expressed immune-related genes of tumors from mice treated with no IR (anti-CTLA4 and IgG) vs. with IR (16 Gy + anti-CTLA4 and 16 Gy + IgG). Fold-change cut-off 0.5, adjusted p-values (Benjamini-Hochberg) < 0.05. (c) Enrichment analysis using NanoString PanCancer Immune Profiling Panel for significant genes (by p-adj value) in IR group (16 Gy + anti-CTLA4 and 16 Gy + IgG). (d) Heatmaps of differentially expressed immune-related genes of tumors from mice treated with anti-CTLA4 vs. IgG and 16 Gy + anti-CTLA4 vs. 16 Gy + IgG. (e) Volcano plots of differentially expressed immune-related genes of tumors from mice treated with anti-CTLA4 vs. IgG and 16 Gy + anti-CTLA4 vs. 16 Gy + IgG. Fold-change cut-off 0.5, p-values (Benjamini-Hochberg) < 0.05. (f) Enrichment analysis using NanoString PanCancer Immune Profiling Panel for significant genes (by p-value) in 16 Gy + anti-CTLA4 group.
